# Spatially incompatible tool use does not induce tactile neglect

**DOI:** 10.3758/s13414-025-03170-y

**Published:** 2025-12-02

**Authors:** Yanick Kloss, Wilfried Kunde

**Affiliations:** Department of Psychology (III), University of Würzburg, Würzburg, Germany

**Keywords:** Goal-directed movements, Motor control, Perception and action

## Abstract

Operating a tool that moves incompatibly with one’s own body invokes conflict between representations of anticipated body-related and environment-related movement consequences. To revolve such conflict, agents might down-regulate (or “neglect”) representations of body-related movement consequences during action preparation and execution. We tested body-related neglect by probing participants’ tactile sensitivity when operating a tool which moved either spatially compatible or incompatible to their operating hand. Across four experiments, we found no evidence for conflict-induced body-neglect. These results suggest that any potential downregulation of body-related movement components does not extend to the processing of tactile stimulation. We discuss these results with respect to applied settings in which incompatibly moving tools are routinely used.

**Supplementary Information:**

The online version contains supplementary material available at 10.3758/s13414-025-03170-y.

## Introduction

When humans move, they produce perceptual changes across multiple modalities. Consider tool use, like moving a cursor with a computer mouse. In this case, movements produce tactile and kinesthetic changes arising from the moving hand as well as visual changes arising from the moving cursor on the screen. There is evidence that the production of such movements becomes increasingly difficult when larger discrepancies exist between these various feedback signals in terms of spatial direction (e.g., Chen & Proctor, 2013; Pfister & Kunde, 2013), time-point (Dignath et al., 2014), or intensity (Kunde et al., 2004). A rather drastic discreprancy is the inversion of the felt and seen movement direction. This inevitably occurs when using, for example, a lever with a pivot, such as in laparoscopic surgery. Here, the operator’s hand needs to move in one lateral direction to shift the tool tip inside the body in the opposite direction, sometimes denoted as ‘fulcrum effect’ (Gallagher et al, 1998). Despite considerable practice, decrements to generate appropriate movements with such tools can remain (Kunde et al., 2007).

The reasons for performance costs of spatially incompatible tool-transformations are likely manifold. However, they may partly arise at the stage of movement planning and initiation, prior to actually moving. This assumption is inspired by the idea that efferent activites are coded in terms of the perceptual changes they have been experienced to bring about. Generating a movement would then require recalling its associated perceptual consequences. In fact, there is considerable evidence for this so called ideomotor approach (see Pfister, 2019; Shin et al., 2010, for reviews).

The problem posed by highly discrepant multimodal effects can be termed *code interference* (Janczyk & Kunde, 2020). To illustrate, imagine (or try yourself) rotating your computer mouse 180°. Now, to move the relevant part of this digital tool (i.e., the cursor) to the right, you must move the hand to the left. The code interference model suggests that the anticipation of the intended rightwards coded environment-related effect (i.e., the visual image of the cursor moving to the right) will also activate overlapping, rightwards coded body-related action effects (e.g., the kinesthetic and tactile feedback of a rightwards hand movement). This occurs because, based on lifelong learning, visual and body-related effects typically move spatially compatible; we usually see and feel our hand moving rightwards simultaneously. The activated body-related code (rightward) will to some extent activate motor patterns linked to these body-related codes (a rightward hand movement), even though a leftward movement is required.

A straightforward solution to this problem would be to downregulate currently task-irrelevant movements codes, thereby preventing code interference. In most tool use situations it is the movement of the tool that is task-relevant, whereas the body-related movement is nominally task-irrelevant. And in fact, there is preliminary evidence for such *body-related* *“neglect.”*

First, agents seem to be largely unaware of what their body is doing while using a tool if it does not correspond precisely to the tool’s visual effect in the environment: when asked to report how they perceived their (invisible) bodily movements during tool use, people do not only fail to detect discrepancies between the body and the tool, but even shift their felt body position towards the seen position of the tool (Debats et al., 2017; Debats & Heuer, 2018; Heuer & Rapp, 2012; Knoblich & Kircher, 2004; Ladwig et al., 2012; Müsseler & Sutter, 2009; Sutter et al., 2008, 2013).

Second, stimulus compatibility effects seem to be primarily driven by environment-related rather than body-related action effects when a lever transforms hands movements into incompatible tool movements (Kunde et al., 2007; Müsseler et al., 2008; Proctor et al., 2004; Sabek et al., 2025). If the environment-related effect is task-irrelevant, this can reverse. However, when the reliability of proprioceptive and tactile information is interfered with, agents once again favor environment-related effects for action control (Sutter & Ladwig, 2012). Thus, they downregulate body-related effect codes in favor of environment-related ones, as is suggested by the body-related neglect hypothesis.

The third and perhaps most striking piece of evidence is provided by a deafferent patient who had lost all tactile and proprioceptive perception after two episodes of sensory polyneuropathy. In a mirror-drawing task, this patient showed no difficulty performing bodily movements spatially incompatible with the seen drawing in the mirror, in stark contrast to neurotypical control subjects (Lajoie et al., 1992). Without any bodily perception, incompatibility costs disappeared, presumably because there no body-related effect codes interfered with environment-related ones. Instead, the patient seemed able to control his actions solely via anticipation of environment-related effects, an interpretation also supported by findings in healthy participants (Kunde & Weigelt, 2005; Mechsner et al., 2001).

Fourth, in unpublished research from our lab, we observed indications for body-related neglect during incompatible tool-transformations. Liesner (2021) applied tactile stimuli to participants’ hands shortly before they initiated a hand movement controlling a cursor on a screen. When participants planned hand movements that would be transformed into spatially incompatible instead of compatible cursor movements, tactile sensitivity was reduced. Yet this effect was small in size and remains unreplicated, raising concerns about its robustness.To summarize, there is preliminary evidence for a downregulation of interfering body-related effect codes when generating visual effects that are incompatible to felt body movements. Here, we tested whether such downregulation manifests as reduced tactile sensitvity. Specifically, we asked whether sensitivity to externally imposed tactile simulation decreases when using incompatibly compared with compatibly moving tools.

Modulations of tactile sensitivity have been reported in several related research fields, but it is fairly difficult to predict whether corresponding phenomena and the explanations behind transfer to (in)compatible tool use that we studied here. For example, tactile sensitivity decreases in an effector during movement planning and execution, a phenomenon known as tactile suppression. One prominent explanation holds that predictions about the somatosensory consequences of the movement attenuate those exact signals to free capacity for unpredictable sensory events (Broda et al., 2020; Juravle et al., 2017). Body-related neglect due to incompatible tool-transformation, as discussed above, might be seen as being superposed on such tactile suppression that appears to generally occur whenever a body limb is supposed to move. Although we do no want to exlude this possibility, we find it difficult to construe haptic neglect with incompatibly moving tools as an amplification of the general phenomenon of tactile suppression. Foremost, we see no obvious reason why the felt movement of the hand is predicted more accurately or more likely with incompatible compared with compatible tool transformations. Moreover, research in the rubber hand illusion suggests that it is sometimes unpredicted somatosensory information that becomes suppressed: To reduce prediction errors (Friston, 2005) arising from the conflict between the visual input from a fake hand and tactile sensations from a real hand, the latter is downweighted in favor of the visual stimulation which comes with higher reliability. This attenuation manifests as reduced sensitivity in the hidden compared with the passive hand (e.g., Rossi Sebastiano et al., 2022). Our ideomotor-inspired model shares the idea of downregulation of conflicting somatosensensory information, though not to reduce prediction errors as in the passive rubber hand illusion but to overcome interference of body-related and tool-related goal codes during planning an active movement.

To explore this issue holds not only theoretical relevance. Consider a surgeon performing minimal-invasive surgery. He or she may engage in body-related neglect to overcome interference when working with an endoscopic tool. However, a surgeon must retain high tactile sensitivity, as it is crucial, for example, to avoid damaging tissue (Westebring-van der Putten et al., 2008). Moroever, tactile stimulation is often used as a kind of warning signal for users in human-machine interaction (Fang et al., 2024; Spence & Ho, 2008). While the constraints on fast responses to such tactile stimulation has been explored to some extent (Tandonnet et al., 2014), the question of how sensitivity to such stimulation is shaped by different tool-transformations has to our knowledge not been studied in depth.

Here, we present four experiments with a shared basic idea. Participants were asked to move a virtual tool in a specified direction, indicated by an imperative stimulus. Shortly before or after movement onset, or during rest (as a baseline), brief tactile stimulation was applied to the operating hand, and we measured tactile sensitivity in various ways. In Experiments 1, 3, and 4, participants were to judge the absence or presence of a tactile stimulus at a fixed location on their active hand, while Experiment 2 asked them to locate an easily detectable stimulus at a left or right position on their active hand. Crucially, in different conditions the tool moved predictably either in a spatially compatible or incompatible way relative to the hand. We expected to observe reduced tactile perceptual performance when planning or executing a hand movement compared with resting. The key question, however, was whether this tactile supression was more pronounced when operating an incompatibly, rather than compatibly, moving tool.

## Experiment 1

The first study followed two goals: first, to replicate the findings by Liesner (2021) in support of the body-related neglect hypothesis, and second, to investigate if body-related neglect observed during action planning persisted during it’s execution.

### Method

A power analysis in RStudio (Version 2023.09.1; RStudio Team, 2023) revealed that 52 participants sufficed to obtain a minimal power of 1 − β = 0.8 to detect a small to medium effect size of *d*_z_ = 0.4 at an error probability of α = 0.05. Data collection was stopped after 65 participants, recruited via Sona systems (https://www.sona-systems.com/), had given informed consent to take part in the experiment. One participant had to be excluded due to failure of the technical equipment; five participants did not finish the experiment; finally, nine participants were excluded because their responses indicated that they could not or did not try to discriminate between the different tactile intensities (see Fig. S2 in the Supplementary Materials for psychometric plots of an exemplary participant). Therefore, 49 eligible datasets (*M*_age_ = 27 years, 36 women) were analyzed. We preregistered to include whether participants had taken part in a pilot experiment as a covariate but omitted it as it showed no meaningful effect (see the Supplementary Materials for an analysis excluding those participants).

#### Materials and apparatus

Participants were placed in front of a 24-in. screen at a standard distance of around 30 inches. They had an electromagnetic solenoid-type stimulator (Dancer Design, St. Helens, Merseyside, UK) attached between thumb and index finger of their active hand. This device converts the frequency and amplitude of an audio signal into vibrations of a small pin. Based on pilot testing, participants received 250 Hz stimulations with a peak-to-peak displacement of the vibrating element across six different levels, ranging from 10.33 to 11.17 µM (based on manufacturer testing; real displacement will depend on participants’ skin properties and tactor placement). The exact positioning of the tactor(s) in all four Experiments is depicted in Fig. S3 in the Supplementary Material.

#### Procedure

The experiment comprised a baseline block as well as a compatible and an incompatible tool-transformation block. In total, each block consisted of at least 144 trials (16 per intensity level, 48 without stimulation). Error trials were repeated at the end of each block to ensure equal cell size.

**Fig. 1:**
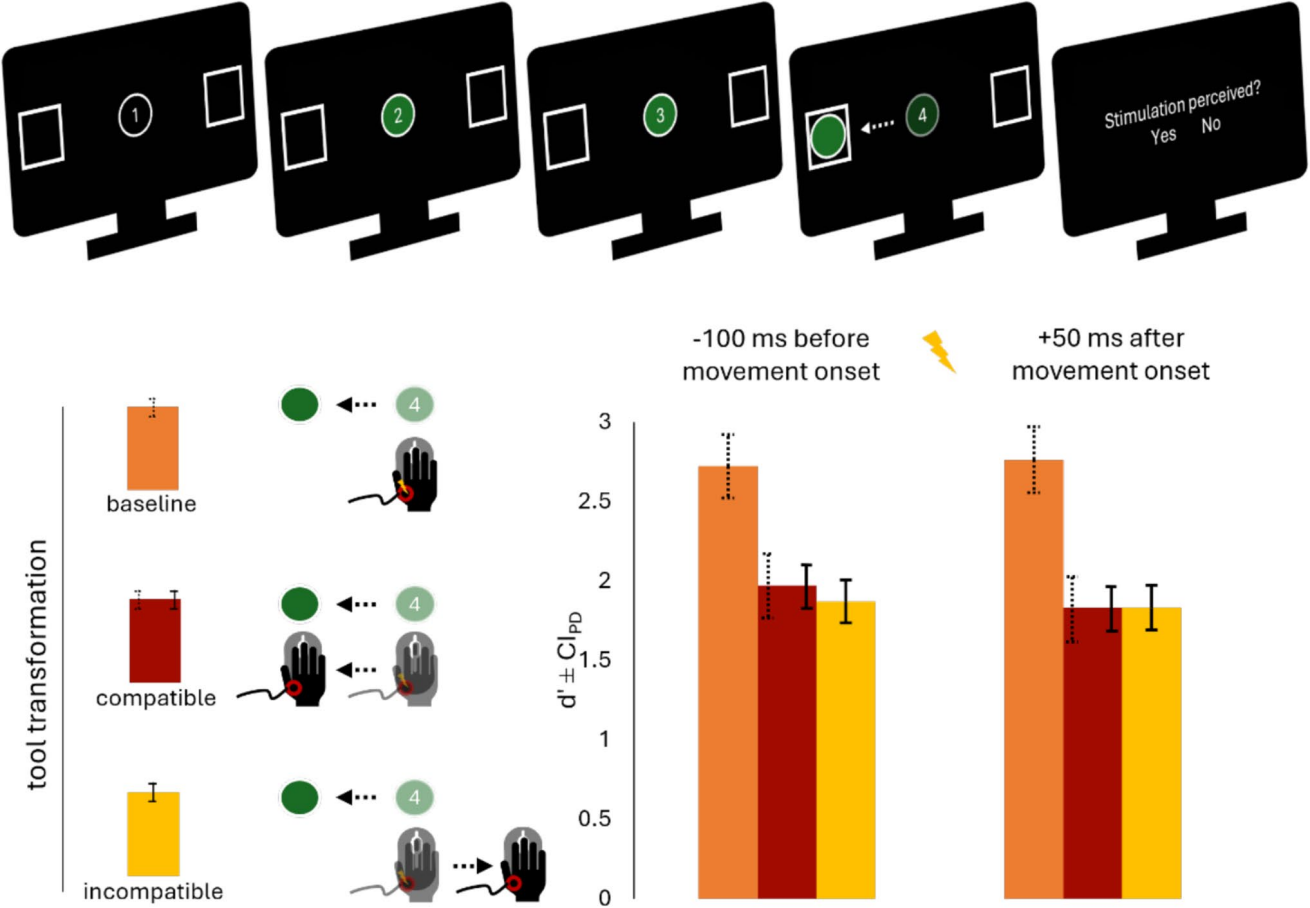
Trial procedure and tactile sensitivity as reflected in* d′ *as a function of tool-transformation condition and stimulation timing in Experiment 1. Dashed error bars represent confidence intervals of the paired differences between the baseline and the compatible condition, while solid error bars represent confidence intervals of the paired differences between the compatible and the incompatible condition (Pfister & Janczyk, 2013). Before movement onset *CI*_*PD*_ _baseline-incompatible_ = 0.19, and after movement onset *CI*_*PD*_ _baseline-incompatible_ = 0.21

Figure 1 illustrates the trial procedure. In the transformation blocks, participants placed their right hand on the computer mouse and viewed two squares at the right and left side of the screen and a circle in the center. These blocks employed the same timed-movement initiation procedure used by Liesner (2021): Following a variable delay (1,000 or 2,000 ms), a visual timer appeared, counting from 1 to 4 at 500 ms intervals. At “2,” the circle turned either green or blue, indicating which square it should be moved into (e.g., “If green, place in the right square”).

Critically, participants were not instructed to move their hand in one way or the other, but to place the circle within either of the two squares to emphasize the relevance of the environment-related effect (Wirth et al., 2016). They received the following instruction to initiate their response: “The movement is not to be initiated right after the color change, but only once the counter has counted to ‘4’. Try to move precisely *with* the ‘4’. Do not react once the ‘4’ has appeared, but when the ‘4’ will appear.” Movements deviating more than 200 ms from the onset of “4” were excluded.

In the compatible block, the circle was moved to the right [/left] by moving the mouse to the right [/left]. In the incompatible block, the circle was moved to the right [/left] by moving the mouse to the left [/right]. Mouse movements on the y-axis were not transformed into circle movements. Participants were only informed about the transformation rule at the start of each block. Moving the target beyond the square or in the wrong direction triggered an error and feedback. In the baseline block, the circle moved independently of the mouse at a speed mirroring subjects’ average response durations in a pilot study employing a similar design to achieve a visual effect similar to that in the two movement conditions. Any mouse movement resulted in an error trial.

In a third of the trials of each block, no stimulation was presented. In the other half, participants received one of six stimulus intensities, either 100 ms before or 50 ms after the onset of “4.” Each intensity/timing combination was presented at least eight times per block, evenly split between left/right target directions.

Participants were randomly assigned to one of two stimulus-effect mappings (green [/blue] → circle to be moved to the right [/left] vs. green [/blue] → circle to be moved to the left [/right]). The order of hand-to-tool-transformation was counterbalanced across participants. Combinations of stimulation timing, required response, and stimulus intensity level were presented in a random order.

After the correct completion of a trial, participants were to indicate if they had perceived a stimulus by pressing one of two keys on the keyboard (1 → yes, 2 → no) with their left hand.

### Results

#### Movement-related performance

Figure 1 summarizes the key results. On average, participants responded correctly to 99.6% of trials in the baseline block (*SD* = 1%; error trials were those where participants did move the mouse in any way whatsoever), and the target moved from the center to either of the two squares within 657 ms. Participants performed better in the compatible than in the incompatible block: They were more accurate (*M* = 85%, *SD* = 11% vs. *M* = 81%, *SD* = 13%), *t*(48) = 2.99, *p* =.004, *d*_z_ = 0.43, more successful at timing their response initiation (*M* = 1 ms after the onset of the four, *SD* = 49 ms, vs. *M* = 10 ms after onset of the four, *SD* = 44 ms), *t*(48) = 2.11, *p* =.040, *d*_z_ = 0.30, and faster at placing the circle in the square (*M* = 529 ms, *SD* = 145 ms, vs. *M* = 723 ms, *SD* = 196 ms), *t*(48) = 10.5, *p* <.001, *d*_z_ = 1.50. On average, the stimulus was applied before participants’ actual movement onset in 44% of all stimulation trials. Figure S1 in the Supplementary Materials visualizes mean stimulation timings relative to movement onset across participants for Experiments 1–4.

A reviewer suggested analyzing performance not only as a function of tool-transformation, but of stimulation as well. The analysis of accuracy in a 2 × 3 analysis of variance (ANOVA) with the factors tool-transformation (compatible vs. incompatible) and stimulation (no stimulation vs. stimulation before movement onset vs. stimulation after movement onset) did not reveal that performance was affected by the presence and timing of a tactile stimulus. A more detailed report and discussion of the results across the four experiments can be found in the Supplementary Materials.

#### Tactile detection

Tactile suppression effects (as reflected in the difference in sensitivity between the baseline and the compatible condition) and body-related neglect effects (as reflected in the difference in sensitivity between the compatible and the incompatible condition) are summarized in Table 1 with corresponding Bayes factors, as are all ANOVA results.

**Table 1:** Tactile suppression and body-related neglect effects and the respective ANOVA results across Experiments 1–4

		Effect												
		Tactile suppression Δbaseline-compatible				Body-related neglect Δcompatible-incompatible								
		Stimulation timing												
		Before movement onset		After movement onset		Before movement onset		After movement onset						
	Measure	*M*	*BF*_10_	*M*	*BF*_10_	*M*	*BF*_10_	*M*	*BF*_10_	ANOVA effect	*df*_num,den_	*F*	*p*	η_p_^2^
Exp. 1	*d′*	0.75	11.1^E+6^	0.93	15.0^E+8^	0.10	0.42	−0.01	0.16	Transformation	2,96	66.69	<.001	.58
										Timing	1,48	2.24	.141	.05
										Interaction	2,96	5.09	.008	.10
Exp. 2	*d′*	1.17	22.2^E+7^	1.22	10.1^E+8^	0.13	0.27	0.05	0.17	Transformation	2,88	63.20	<.001	.59
										Timing	1,44	2.61	.113	.06
										Interaction	2,88	0.28	.755	<.01
Exp. 3	*d′*					0.04	0.34	−0.03	0.30	Transformation	1,52	0.01	.909	<.01
										Timing	1,52	12.42	<.001	.19
										Interaction	1,52	3.66	.061	.07
	Detection thresholds					−0.18	0.50	0.07	0.33	Transformation	1,42	0.14	.712	<.01
										Timing	1,42	4.53	.039	.10
										Interaction	1,42	3.69	.062	.08
Exp. 4	*d*′					0.02	0.16	−0.01	0.16	Transformation	1,49	<0.01	.974	<.01
										Timing	1,49	0.02	.877	<.01
										Interaction	1,49	0.51	.477	<.01
	Detection thresholds					−0.16	0.41	0.03	0.18	Transformation	1,37	0.52	.478	.01
										Timing	1,37	0.04	.840	<.01
										Interaction	1,37	2.02	.164	.05

Tactile suppression means were computed by subtracting mean d’ in the compatible condition from the baseline condition. Body-related neglect means were computed by subtracting tactile sensitivity means (*d′* or detection thresholds) in the incompatible condition from the compatible condition. Positive values for *d′* and negative values for detection thresholds indicate the existence of the respective effect. All tactile suppression effects were highly significant in Experiments 1 and 2, while no body-related neglect effects were significant in Experiments 1–4. All Bayes Factors were computed with a prior distribution of $\frac{\sqrt{2}}{2}$. Greenhouse–Geisser correction was used to account for sphericity violations in all four experiments

Our design aimed at providing data for the analysis of two measures of tactile sensitivity. Because we could compute reliable detection thresholds only for a small number of subjects (see Table S1 in the Supplementary Materials), we focused on statistics within the signal detection theory framework and computed the sensitivity measure *d*′ and the response bias measure c using the *psycho* package in R (Macmillan, 2001). All trials without any stimulation constituted *Noise* and all trials with stimulation, no matter the intensity, constituted *Signal* trials. When there were no observations within a cell, we added 0.5 while subtracting 0.5 from the respective second signal/noise cell (Kadlec, 1999).

We subjected mean *d*′ to a 3 × 2 repeated-measures ANOVA, with the factors tool-transformation condition (baseline vs. compatible vs. incompatible) and stimulation timing (before vs. after movement onset). Greenhouse–Geisser correction was used to account for sphericity violations. Since there was extremely little variation in the number of trials in the baseline condition, we did not enter this as a covariate despite having preregistered to do so. The ANOVA revealed a main effect of tool-transformation condition, *F*(2,96) = 66.69, *p* <.001, η_p_^2^ =.58 and an interaction effect, *F*(2,96) = 5.09, *p* =.008, η_p_^2^ =.10. We computed pairwise post hoc *t* tests to compare *d*′ at the different stimulation time points in the three blocks. Note that all *p* values are Bonferroni adjusted. D prime (*d*′) was significantly larger in the baseline block compared with both compatible and incompatible tool-transformation blocks at both stimulation timepoints (all *p*s <.001). D prime was descriptively larger in compatible compared with incompatible trials before movement onset (1.97 vs. 1.87), *t*(48) = 1.46, *p* =.456, *d*_z_ = 0.21, *BF*_10_ = 0.42 (prior distribution of $\frac{\sqrt{2}}{2}$, as for all Bayes factors reported in this manuscript), but not after movement onset (1.83 vs. 1.83), *t*(48) = 0.01, *p* =.999, *d*_z_ < 0.01, *BF*_10_ = 0.16.

We further analyzed the response criterion c (see Table S1 in the Supplementary Materials for all ANOVA effects). The ANOVA revealed a main effect of tool-transformation condition, *F*(2,96) = 11.59, *p* <.001, η_p_^2^ =.19 and an interaction, *F*(2,96) = 5.09, *p* =.008, η_p_^2^ =.10. Post hoc *t* tests indicated that the main effect of transformation condition was driven by the baseline condition, with smaller c compared with both movement conditions before and after movement onset (all *p*s <.008; for the comparison of compatible and incompatible trials, all *p*s >.639).

We explored Hartigan’s Dip Statistic (Freeman & Dale, 2013; Hartigan & Hartigan, 1985; Pfister et al., 2013) to test for a bimodal distribution of d’ to investigate if some participants explicitly recoded instructions to map stimuli not to the effect on the screen, but to their bodily movement (see the Discussion for a detailed explanation of this account), but found no evidence of such, *D* = 0.04, *p* =.915.

### Discussion

In line with previous findings (e.g., Colino & Binsted, 2016; Juravle et al., 2017; Voss et al., 2008), tactile sensitivity on an effector was clearly reduced both shortly before and after movement initiation compared with a baseline condition without action planning. The response criterion also suggested that participants required less evidence to report a tactile stimulus in the baseline condition. However, this was not reflected in another measure of response criterion (beta), nor was it replicated in subsequent experiments, suggesting it was likely a spurious observation.

However, we did not observe reduced tactile sensitivity when movements produced visual effects that were spatially incompatible, as opposed to compatible, with the agent’s body movements. This was despite clear indications that participants struggled with the incompatible transformation of hand movements into movements of the digital tool, as shown by slower response times and reduced accuracy.

The design specifically aimed to ensure that individuals anticipated the environment-related effects of their movements. However, we cannot rule out the possibility that participants explicitly recoded the instructions, mapping each color stimulus not to the environment-related effect, but to the hand movement needed to achieve this in the current block (e.g., replacing *When the circle is green, place it* *in the right square* with *When the circle is green, move hand to the left*). Such a recoding strategy has been proposed to explain reductions in spatial Simon effects when the task-irrelevant stimulus dimension is incompatible with the environment-related action effect but compatible with the body-related action effect (Sutter & Ladwig, 2012). In our case, it might have eliminated the need to downregulate body-related effect codes that might otherwise have been activated during anticipation. However, the differences observed in response times, accuracy, and response durations do not support this assumption. If participants had indeed tried to recode the instructions and ignore the environment-related effect, they were not very successful in doing so. Still, we reasoned that if some participants did in fact apply such an explicit recoding strategy while others did not, differences between sensitivity during compatible and incompatible tool-transformations should be distributed bimodally (Freeman & Dale, 2013). This was not the case.

To further investigate why we did not find body-related neglect, we sought to test different potential causes in three additional experiments: Experiment 2 scrutinized if the supposed neglect of body-related effect codes is restricted to those very dimensions where body- and environment-related effect features are actually incompatible. Experiment 3 addressed methodological limitations concerning power, outliers, and stimulus settings. Finally, Experiment 4 tested a range of explanations regarding the specificity of the supposed neglect to certain effect dimensions and locations, participants’ recoding strategies, and habituation effects.

## Experiment 2

First, we explored the idea that the neglect of body-related action effects that are in one dimension incompatible with environment-related action effects is confined to codes related to that very dimension. Specifically, the spatial nature of the incompatibility in Experiment 1 might have induced a neglect of spatial codes related to the agent’s body, but not (or only to a weaker extent) a neglect of intensity-related codes, as assessed by the tactile stimulus detection task. Therefore, Experiment 2 employed the same main task to induce neglect of spatial body-related effect codes, but a different method to measure it specifically. Instead of detecting the presence of tactile stimuli with near-threshold intensity levels, participants now localized a clearly detectable stimulus applied to the right or left side of their active hand.

### Methods

As preregistered, we stopped data collection once we had gathered sufficient evidence for the null hypothesis that there was in fact no difference between sensitivity during compatible and incompatible tool-transformations. Up to this point, 56 individuals, recruited via Sona systems, had given informed consent to participate in the study. Three of them did not complete the experiment; eight were excluded because their performance in discriminating the stimulation location in the baseline condition did not differ significantly from chance at an α =.025. We report results for the remaining 45 participants (39 women, *M*_age_ = 24 years).

#### Materials and apparatus

We used the same setup as in Experiment 1. However, instead of one, we now placed two tactors horizontally next to each other in the center of the back of participants’ active, right hand. The tactors’ vibrating elements had a distance of 2 cm. Tactors vibrated at a frequency of 250 Hz and produced peak-to-peak displacements of the vibrating element of 13.33, 16.67, or 20 µM.

#### Procedure

A pretest ensured that participants perceived the stimulation at both tactor locations as equally strong. Then, the experiment followed the same design as Experiment 1, except that the stimulations before and after movement onset differed not in their perceived intensity, but in their location. Further, there were no trials without any stimulation, but participants knew that they would always be stimulated by either of the two tactors. After each trial, participants were to judge if they perceived the stimulation through the right or the left tactor.

### Results

#### Movement-related performance

**Fig. 2:**
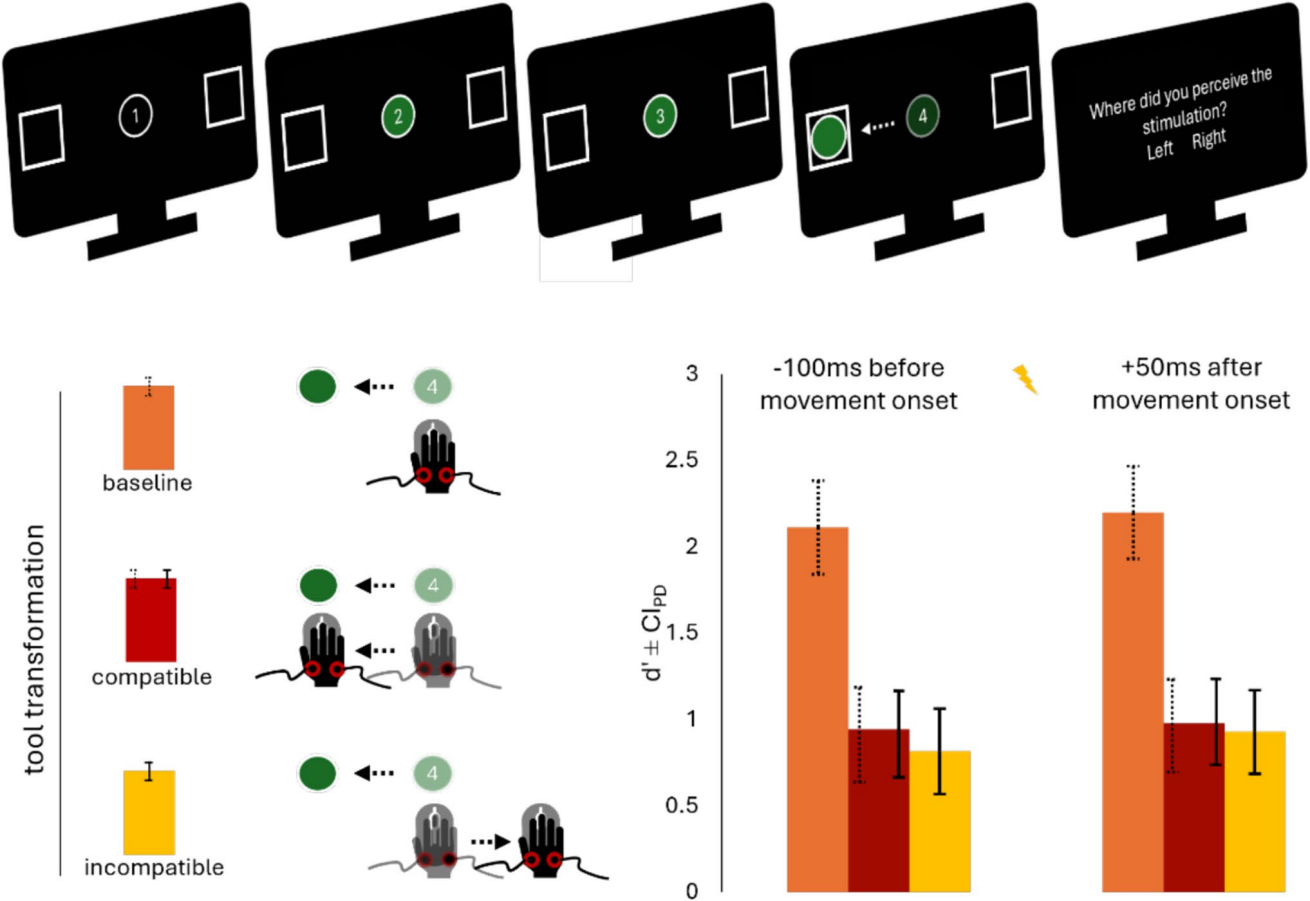
Trial procedure and tactile sensitivity as reflected in *d′* as a function of tool-transformation condition and stimulation timing in Experiment 2. Dashed error bars represent confidence intervals of the paired differences between the baseline and the compatible condition, while solid error bars represent confidence intervals of the paired differences between the compatible and the incompatible condition (Pfister & Janczyk, 2013). Before movement onset *CI*_*PD*_ _baseline-incompatible_ = 0.29, and after movement onset *CI*_*PD*_ _baseline-incompatible_ = 0.34

Figure 2 summarizes the key results. On average, participants responded correctly to 99.6% of trials in the baseline block (*SD* = 1%; note that they were not to respond at all in this condition, but that the stimulus moved on its own from the center into the respective square within 634 ms). In the compatible block, accuracy was descriptively larger compared with the incompatible block (*M* = 81%, *SD* = 11% vs. *M* = 79%, *SD* = 10%), *t*(44) = 1.47, *p* =.149, *d*_*z*_ = 0.22. Participants also performed descriptively better at timing their response initiation (*M* ≤ 1 ms before, *SD* = 48 ms vs. *M* = 3 ms after onset of the four, *SD* = 45 ms), *t*(44) = 0.58, *p* =.563, *d*_z_ = 0.09, and they were significantly faster at moving the circle from the center into the respective square (*M* = 615 ms, *SD* = 169 ms vs. *M* = 849 ms, *SD* = 231 ms), *t*(44) = 9.47, *p* <.001, *d*_z_ = 1.41. On average, the stimulus was applied before participants’ actual movement onset in 43% of all trials.

Again, we also explored if accuracy was affected by the timing of the tactile stimulation. We computed the same ANOVA as in Experiment 1 that now revealed a main effect of stimulation timing, *F*(1,44) = 9.18, *p* =.004, η_p_^2^ =.17. In compatible trials, accuracy was larger when participants received a tactile stimulus shortly before initiating their movement, *t*(45) = 3.68, *p* <.001, *d*_z_ = 0.55.

#### Tactile detection

Left tactor trials were classified as *Signal*, right as *Noise*. Per condition, we classified *Hits* when a participant located the stimulus correctly on the left side, *Misses* when they located it incorrectly on the right, *False Alarms* when they located it incorrectly on the left, and *Correct Rejections* when they located it correctly on the right.

We subjected mean *d*′ to a 3 × 2 repeated-measures ANOVA with the factors tool-transformation condition (baseline vs. compatible vs. incompatible) and stimulation timing (before vs. after movement onset). The ANOVA revealed only a main effect of tool-transformation condition, *F*(2,88) = 63.20, *p* <.001, η_p_^2^ =.59. We computed pairwise post hoc *t* tests to compare *d*′ at the different stimulation time points in the three blocks. The results mirrored those of Experiment 1: *d*′ was significantly larger in the baseline compared with both tool-transformations before and after movement onset (all *p* <.001). D prime was only descriptively larger in compatible compared with incompatible trials, both before (0.94 vs. 0.82), *t*(44) = 1.04, *p* =.915, *d*_z_ = 0.16, *BF*_10_ = 0.27, and after movement onset (0.98 vs. 0.93), *t*(44) = 0.40, *p* =.999, *d*_z_ = 0.06, *BF*_10_ = 0.17.

For c, the ANOVA revealed no effects (all *p*s >.446), and none of the post-hoc tests were significant (all *p*s >.660).

To explore if the spatial nature of the localization task interacted with the spatial compatibility of the tool-transformations, we compared *d*′ across Experiments 1 and 2. A 2 × 2× 2 mixed ANOVA with the within-factors tool-transformation condition (baseline vs. compatible vs. incompatible) and stimulation timing (before vs. after movement onset) and the between-factor experiment (Experiment 1 vs. 2) revealed not only a main effect of tool-transformation, *F*(1.77,162.41) = 533.43, *p* <.001, η_p_^2^ =.58, but also a main effect of experiment, *F*(1,92) = 30.52, *p* <.001, η_p_^2^ =.25, and an interaction effect of experiment with stimulation timing, *F*(1,92) = 4.86, *p* =.030, η_p_^2^ =.05, and, crucially, tool-transformation, *F*(1.77, 162.41) = 4.05, *p* =.024, η_p_^2^ =.04. D prime was significantly larger in Experiment 1 compared with Experiment 2 across all tool-transformations and stimulation timings (all *p*s ≤.002). However, effect sizes were considerably larger for the two tool-transformation conditions (all 1.06 ≥ *d* ≥ 0.85) compared with the baseline condition (before movement onset *d* = 0.61 and after movement onset *d* = 0.57). In fact, the differences in mean *d*′ between the baseline and compatible condition were significantly larger in Experiment 2 than in Experiment 1 both before, *t*(82) = 2.69, *p* =.009, *d*_*z*_ = 0.58, and after movement onset, *t*(82) = 2.07, *p* =.042, *dz* = 0.44.

### Discussion

Experiment 2 aimed to test whether the proposed body-related neglect during incompatible tool-transformations is restricted to, or particularly pronounced for, effect codes in the specific dimension where incompatibility between body- and environment-related codes exists. However, the results mirrored those of Experiment 1: The ability to discriminate the location of a tactile stimulus was strongly reduced on the effector shortly before and after movement onset, but this reduction did not depend on the compatibility between body- and environment-related action effects.

Although participants performed exactly the same effector movements in both experiments, the general tactile suppression effect was larger in Experiment 2. This was driven by especially poor performance in the stimulus location judgment in the compatible condition, while baseline d’ were not affected as strongly. In other words, participants performed only slightly worse at locating the stimulus as at judging its’ presence or absence at rest, but the former was affected much more strongly by preparing and executing a rightwards/leftwards movement. This pattern aligns with previous observations that tactile suppression is highly specific to the perceptual codes occupied by the anticipated effects (Fuehrer et al., 2022). In our case, anticipating spatial visual, proprioceptive, and tactile effects resulted in stronger suppression of spatial than intensity-based codes.

## Experiment 3

Next, we tested whether we failed to find a body-related neglect effect in Experiments 1 and 2 because of issues related to power, data quality, and stimulation settings. Due to the high exclusion rate, the sample size of Experiment 1 was sufficient to detect a small to medium effect size of *d*_*z*_ =.40 only with a power of 1 − β = 0.67. Exclusions were mostly caused by two factors: high error rates in the two movement conditions, and inappropriate intensity ranges for a large share of participants.

Therefore, Experiment 3 differed from Experiment 1 in three major ways: First, we replaced the timed movement with a simpler Wait-Go paradigm to reduce the amount of errors; Second, we removed the baseline condition to receive stimulus intensities appropriate for the two movement conditions; Third, we employed a pretest to identify an appropriate intensity range for each participant.

### Method

We stopped data collection after 65 individuals, recruited via Sona systems, had given informed consent to participate in the study. We excluded five participants because they performed accurately in less than 60% of trials of at least one of the two blocks, and three participants because their judgments were unrelated to stimulation intensity. Finally, one participant was excluded because they had less than six correct trials for each intensity, and another participant was excluded because their psychometric responses indicated that the tactile stimulation device failed to apply stimulation at some point in the experiment. We report results for the remaining 55 participants (40 women, *M*_age_ = 26). For each analysis, we excluded participants if one of their cell means for the variable under investigation deviated more than 2.5 standard deviations from the respective group cell mean.

#### Materials and apparatus

We used the same setup as in Experiment 1. The tactile stimulator produced peak-to-peak displacements between 6.67 µM and 30 µM, based on participants’ optimal intensity range. The lowest intensity level was always 6.67 µM, with seven higher levels spaced equally within each range. The highest intensities across ranges varied from 9 to 30 µM. Table S2 in the Supplementary Material summarizes the ranges.

#### Procedure

Participants first completed a pretest to determine their optimal intensity range. They moved the computer mouse freely to the right or left after a fixation cross was replaced by “GO,” without receiving visual feedback to avoid practice effects. Shortly before or after movement initiation, they received the maximum intensity of each range (14 trials per range) or no stimulation (48 trials). After each movement, they reported whether they felt a stimulus. We selected the lowest range in which the maximum intensity was detected in more than 78% of correct trials. If no range met this criterion, the highest range was used.

**Fig. 3:**
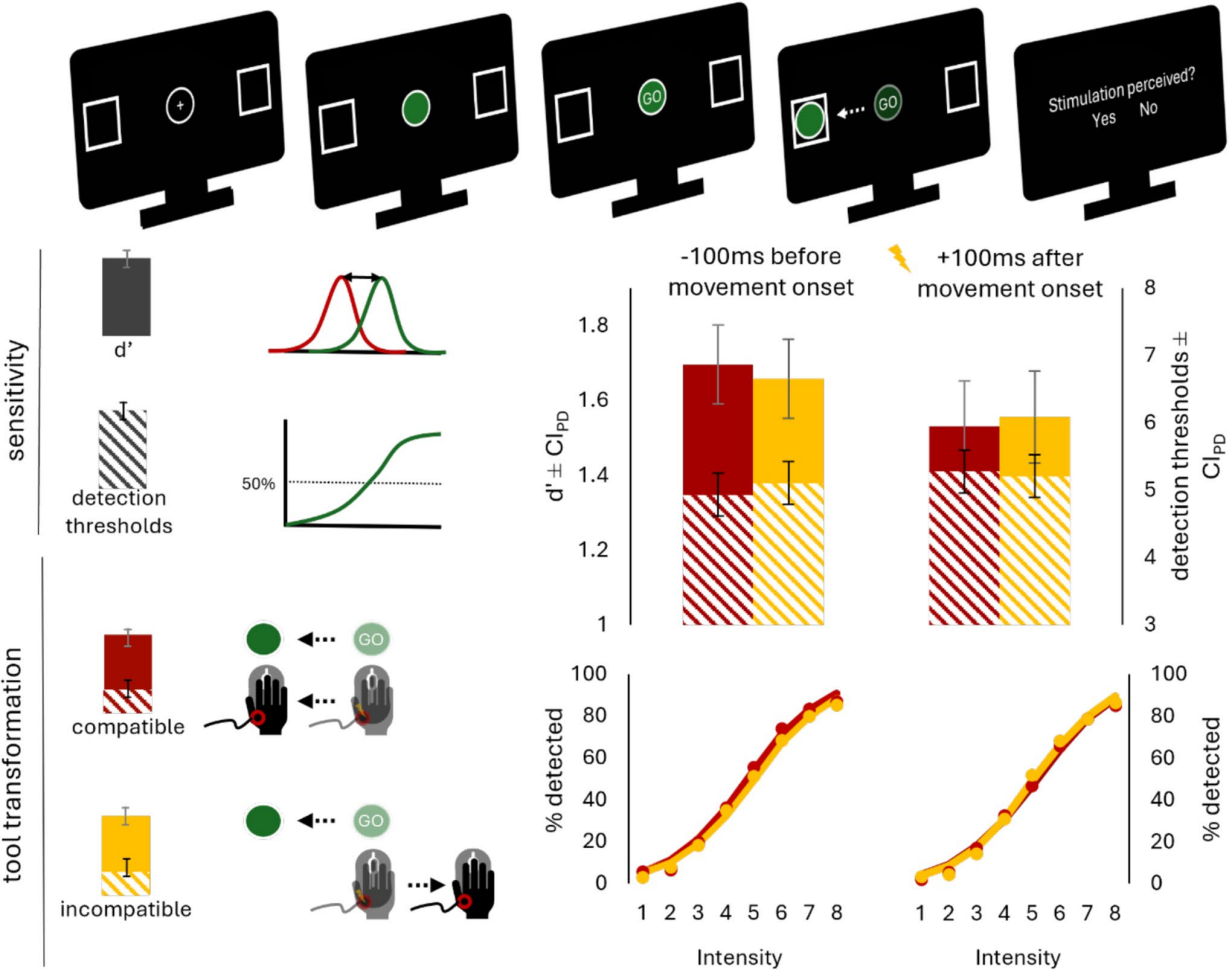
Trial procedure, *d′*, detection thresholds, and observed and predicted detection rates as a function of tool-transformation condition and stimulation timing in Experiment 3. Error bars represent confidence intervals of paired differences (Pfister & Janczyk, 2013)

The main experiment consisted of one compatible and one incompatible block, each preceded by 15 practice trials. In each block, participants completed 18 trials per intensity level and stimulation timing, plus 96 noise trials without stimulation. Figure 3 illustrates the procedure: Participants viewed two squares on either side of the screen and a central circle. After 500 ms of fixating a cross inside the circle, it turned green or blue to indicate the response direction. They were instructed to withhold their response until “GO” appeared after 1,000 ms. In 75% of trials, they received a tactile stimulus on their active hand either 100 ms before or after movement initiation. Movement initiation time was estimated based on the mean of the previous ten correct trials per condition.

### Results

#### Movement-related performance

Figure 3 summarizes the key results. Performance was better in the compatible than in the incompatible block in terms of accuracy (*M* = 87%, *SD* = 6% vs. *M* = 82%, *SD* = 8%), *t*(54) = 6.10, *p* <.001, *d*_z_ = 0.82, response times (*M* = 291 ms, *SD* = 42 ms vs. *M* = 304 ms, *SD* = 32 ms), *t*(52) = 2.24, *p* =.029, *d*_z_ = 0.31, and response durations (*M* = 536 ms, *SD* = 132 ms vs. *M* = 800 ms, *SD* = 187 ms), *t*(52) = 17.7, *p* <.001, *d*_z_ = 2.43. On average, participants were stimulated 104 ms before movement onset (53% of all stimulation trials) and 115 ms after movement onset in the respective conditions.

The analysis of response times and accuracy did not reveal that performance was affected by the presence and timing of a tactile stimulus. A more detailed report can be found in the Supplementary Material.

#### Signal detection theory statistics

We computed d’ the same way as in Experiment 1 and subjected them to a 2 × 2 repeated-measures ANOVA with the factors tool-transformation condition (compatible vs. incompatible) and stimulation timing (before vs. after movement onset). The ANOVA revealed only a main effect of stimulation timing with larger *d*′ before than after movement onset, *F*(1,52) = 12.42, *p* <.001, η_p_^2^ =.19. We computed pairwise post hoc *t* tests to compare *d*′ at the two stimulation time points in both blocks. It was only descriptively larger in the compatible compared with the incompatible block before movement onset (1.70 vs. 1.66), *t*(52) = 0.72, *p* =.475, *d*_z_ = 0.16, *BF*_10_ = 0.34, while the opposite was the case after movement onset (1.53 vs. 1.56), *t*(52) = 0.41, *p* =.685, *d*_z_ = 0.02, *BF*_10_ = 0.30.

The ANOVA for the response criterion c revealed only a main effect of stimulation timing, *F*(1,52) = 10.79, *p* =.002, η_p_^2^ =.17, with smaller c before than after movement onset.

#### Detection thresholds

In addition to *d*′, we computed psychometric functions per subject and condition using the *quickpsy* package in R. Detection thresholds represent the intensity level at which an individual is expected to detect the tactile stimulus in 50% of the times. Note that each level does not correspond to one fixed peak-to-peak displacement of the tactor, but to its ordinal position within each subject’s range. Table S2 includes the number of participants assigned to each range. Figure 3 shows average detection rates and psychometric functions fitted over all subjects. The analysis mirrored that of d’. There was only a main effect of stimulation time, as detection thresholds were smaller before than after movement onset, *F*(1,42) = 4.53, *p* =.039, η_p_^2^ =.10. They were only descriptively smaller in the compatible compared with the incompatible block before movement onset (4.93 vs. 5.11), *t*(42) = 1.12, *p* =.267, *d*_z_ = 0.17, *BF*_10_ = 0.50, while the opposite was the case after movement onset (5.28 vs. 5.21), *t*(42) = 0.45, *p* =.658, *d*_z_ = 0.07, *BF*_10_ = 0.33.

### Discussion

Experiment 3 aimed to rule out the possibility that we underestimated the body-related neglect effect in the two previous experiments due to issues with power and stimulus settings. Despite having presented subjects with individually customized stimulation intensities and increased sample size, we failed to observe a reduction in tactile sensitivity when environment-related action effects were spatially incompatible with effects related to the environment. Tactile sensitivity was only descriptively reduced before movement onset while planning incompatible tool-transformations.

## Experiment 4

The final experiment aimed to maximize the descriptive differences observed before by implementing four major changes: First, recall that we had been attempting to measure the extent of body-related neglect by testing sensitivity for tactile stimuli. However, in the previous design, the potentially interfering body-related effects were primarily proprioceptive in nature. When moving the hand placed on a computer mouse to the right or left, the tactile experience on the hand does not change dramatically. Presumably, participants had little need to neglect tactile effect codes, focusing instead on proprioceptive ones – something our tactile detection tasks may have failed to capture. To address this, Experiment 4 introduced tactile action effects that participants could use to represent their action (Sabek et al., 2025).

Second, we previously discussed the possibility that participants explicitly recoded the instructions, redirecting action goals from the environment to the body. To further discourage the use of this strategy, we increased the number of response options, making such recoding more difficult.

Third, tactile suppression has been shown to be greatest in close proximity to the action effects (Broda et al., 2020). Therefore, we increased the spatial proximity between the potentially neglected action effect in the body and the location of the tactile test stimulus.

Finally, exploratory analyses of Experiment 3 suggested that the difference in sensitivity between incompatible and compatible trials was largest at the beginning of the block. To account for this, we replaced the blockwise with a trialwise manipulation of action-effect compatibility.

Additionally, we improved our set up in two ways to increase compatibility costs in motor performance: First, we presented the visual effect in the horizontal plane, matching the plane in which body movements were performed. Second, we occluded participants’ view of their hand movements to ensure that the only visible action effect was the one they aimed to produce on the screen.

### Method

We stopped data collection after 65 individuals, recruited via Sona systems, had given informed consent to participate in the study. We excluded three participants because they did not finish the experiment, seven because they performed accurately in less than 60% of trials of at least one of the two tool-transformation conditions, and two because their detection judgments showed no clear relationship between intensity level and detection probability, indicating that they did not base their judgments on the actual stimulation. We report results for the remaining 53 participants (40 women, *M*_age_ = 26 years). For each analysis, we excluded participants if one of their cell means for the variable under investigation deviated more than 2.5 standard deviations from the respective group cell mean. For the analysis of detection thresholds, we excluded participants if they had less than four observations per intensity and if their thresholds were outside the range of intensities presented.

#### Materials and apparatus

Participants sat in front of an open-sided wooden cubicle (54 × 36 cm) placed on a desk. On the bottom, we installed a circular 3D-printed Polylactide structure with concentric ridges and grooves. Each ridge had a rectangular cross-section (2 mm height and width), separated by grooves of equal width. At the center was a raised area (20 mm diameter), and the total diameter was 360 mm. Participants placed their right hand inside the cubicle and their left hand on its left side to press response buttons. Figure S4 in the Supplementary Information depicts the setup and a corresponding visual image of a trial.

A video projector attached to a metal construction 100 cm above the box projected the experiment onto its’ upper surface. The entire visual image had a diagonal of 55 cm. Below the projector, a motion tracking device (3D Guidance TrakStar, Waterloo, Ontario, Canda) tracked the position of a sensor attached to participants’ right index fingernail. We also attached the same tactile stimulation device used in Experiments 1–3 to the inside of the proximal phalanx of that finger.

The tactor vibrated at 250 Hz, producing peak-to-peak displacements between 6.67 µM and 40.27 µM, depending on participants’ optimal intensity range. The lowest intensity in each range was 6.67 µM; the highest ranged from 9 to 30 µM. Each range had eight equally spaced intensity levels. Table S3 summarizes all intensity ranges and the number of subjects assigned to each.

#### Procedure

Participants were instructed to keep their right index finger in contact with the ridged surface throughout the experiment. Thus, hand movements always produced tactile action effects spatially compatible with proprioceptive feedback, but either compatible or incompatible with visual feedback.

Again, a pretest determined participants’ optimal intensity range. Participants moved their finger after a “GO” cue until it was replaced by “STOP,” receiving no movement feedback to avoid practice effects. Shortly before or after movement initiation, they received the highest intensity level of each range (8 trials per range) or no stimulation (8 trials). After completing a movement, they reported the presence or absence of stimulation using the left-hand buttons. We selected the range where the maximum intensity was detected in more than 74% of correct trials and chose the range two units higher for the main task. If no range met this criterion, the highest range was used.

**Fig. 4:**
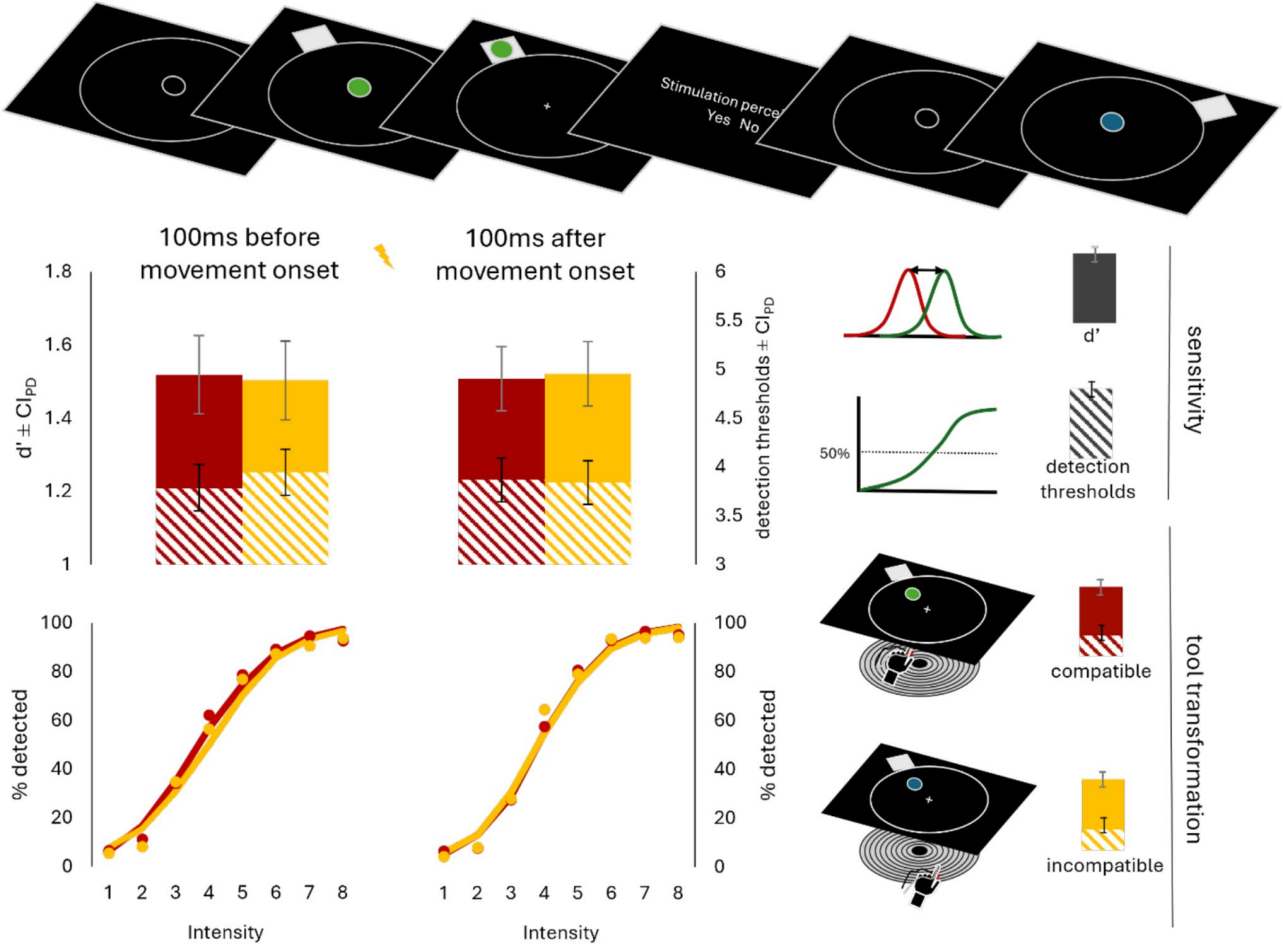
Trial procedure, *d′*, detection thresholds, and observed and predicted detection rates as a function of tool-transformation condition and stimulation time in Experiment 4. Error bars represent confidence intervals of paired differences (Pfister & Janczyk, 2013)

In the main task (Fig. 4), each trial began with a large and a small circle displayed. After 1,000 or 1,500 ms, the small circle at the center turned green or blue, indicating the transformation condition. Participants were instructed: “If the circle is green [/blue], it moves 1-to-1 with your finger. If blue [/green], it moves 180° opposite to your finger.”

When participants’ index finger was positioned at the exact center of the structure, the circle was projected directly above it in both conditions. In the incompatible condition, the circle moved in the opposite direction. For example, moving the finger 10 cm at 90° resulted in the circle moving 10 cm at 270°.

Upon the color change, a white square appeared at one of eight positions around the large circle. Participants had to move the small circle into the square within 1,000 ms, moving towards it (compatible) or away from it (incompatible). Movements exceeding a small margin or moving in the wrong direction were registered as errors (see Fig. S5). Holding the circle in the square for 500 ms completed the trial. As in previous experiments, overshooting the square resulted in an error. Participants then indicated whether they detected a stimulus using the buttons, followed by moving the small circle back to the center to start the next trial.

Tactile stimulation occurred shortly before or after movement initiation in 89% of trials. We estimated movement initiation time based on the mean of the previous ten correct trials (including 28 practice trials, 14 per condition). Until ten correct trials were completed, stimulation timing was based on Experiment 3 averages. Stimulation occurred 100 ms before or after the estimated response time.

The stimulus-response mapping was counterbalanced across participants. Compatible and incompatible trials were presented randomly, as were target positions. In total, participants faced 320 signal trials and 40 noise trials in the main task.

### Results and discussion

#### Movement-related performance

Figure 4 summarizes the trial procedure and key results. Performance was better in compatible than in incompatible trials in terms of response times (*M* = 639 ms, *SD* = 117 ms vs. *M* = 669 ms, *SD* = 99 ms), *t*(51) = 5.46, *p* <.001, *d*_*z*_ = 0.76, and response durations (*M* = 467 ms, *SD* = 186 ms vs. *M* = 621 ms, *SD* = 284 ms), *t*(51) = 10.4, *p* <.001, *d*_*z*_ = 1.44, but not accuracy (*M* = 86%, *SD* = 7% vs. *M* = 86%, *SD* = 6%), *t*(50) = 0.83, *p* =.411, *d*_*z*_ = 0.12. On average, participants were stimulated 94 ms before movement onset (51% of all stimulation trials) and 103 ms after movement onset in the respective conditions.

Again, we analyzed response times also as a function of tactile stimulation (timing), but did not find any effects (see the Supplementary Material for a more detail report). The ANOVA on accuracy did however reveal an interaction between compatibility and stimulation, *F*(2,88) = 11.50, *p* <.001, η_p_^2^ =.21. In compatible tool-transformation trials, accuracy was significantly reduced when a stimulus was applied shortly before movement onset compared with trials where it was applied after movement onset, *t*(47) = 4.11, *p* <.001, *d*_*z*_ = 0.59, and trials where participants received no stimulation at all, *t*(47) = 3.63, *p* =.002, *d*_*z*_ = 0.52. These results could be replicated when categorizing the stimulation timing based on the anticipated movement onset, but they stand in contrast to the results in Experiment 1 and should be therefore taken with great caution.

#### Signal detection theory statistics

We computed *d*′ and c the same way as in Experiments 1 and 3 and subjected them to a 2 × 2 repeated-measures ANOVA with the factors tool-transformation condition (compatible vs. incompatible) and stimulation timing (before vs. after movement onset). The ANOVA for *d*′ revealed no significant effects (all *p*s >.477). We computed pairwise post hoc *t* tests to compare *d*′ at the two stimulation time points in both blocks. Once more, *d*′ was only descriptively larger in the compatible compared with the incompatible block before movement onset (1.52 vs. 1.50), *t*(49) = 0.30, *p* =.767, *d*_*z*_ = 0.04, *BF*_10_ = 0.16, while the opposite was the case after movement onset (1.51 vs. 1.52), *t*(49) = 0.30, *p* =.766, *d*_*z*_ = 0.04, *BF*_10_ = 0.16.

None of the ANOVA effects were significant for c (all *p*s >.339), neither were the post hoc *t* tests (all *p*s >.291).

#### Detection thresholds

Detection thresholds were computed the same way as in Experiment 3. Figure 4 shows average detection rates and psychometric functions fitted over all subjects. The analysis mirrored that of *d*′. None of the ANOVA effects were significant (all *p*s >.164). They were only descriptively smaller in the compatible compared with the incompatible block before movement onset (3.79 vs. 3.95), *t*(38) = 1.36, *p* =.181, *d*_*z*_ = 0.22, *BF*_10_ = 0.41, while the opposite was the case after movement onset (3.87 vs. 3.84), *t*(38) = 0.23, *p* =.819, *d*_*z*_ = 0.04, *BF*_10_ = 0.18.

Experiment 4 incorporated a series of changes to maximize descriptive differences in tactile sensitivity. However, the results aligned with those of the previous experiments. While tactile sensitivity was descriptively lower during the planning of incompatible tool-transformations across all four studies, all Bayes factors were < 1, indicating more evidence against the body-related neglect hypothesis than for it. Further, the descriptive difference did not increase when we specifically tested the neglect of effect codes in the very dimension incompatibility had been induced in, nor was it larger when we introduced incompatibility in the tactile domain.

## General discussion

Across four experiments, we tested the idea that spatially incompatible tool-transformations come with reduced tactile sensitivity compared with compatible tool-transformations as a means to overcome conflict between body-related and tool-related codes during movement production. Therefore, we applied tactile stimulation to participants’ hands at different points in time relative to movement onset, targeting processes related to action execution as well as action initiation in Experiments 1 and 2 and likely also action planning in Experiments 3 and 4. In none of the experiments did we find evidence supporting this hypothesis. We failed to do so despite clear indications for code interference, reflected in increased action production times and error rates.

We consistently observed that having to produce an action reduced tactile sensitivity, a finding that might reflect tactile suppression (Juravle et al., 2017) or general dual task costs (perceiving while planning an action). Yet this reduction in tactile sensitivity was not modulated by the compatibility of the movement’s upcoming and intended tool movements. We believe this to be an important finding, especially in light of the various observations that suggest such body-related neglect, as discussed in the introduction.

Virtual tools like a manually controlled cursor can extend peripersonal space towards the position of the manipulated tool (Bassolino et al., 2010). Moreover, such tools can be experienced as belonging to the own body (Ma & Hommel, 2015). Both phenomena are supposed to result from multisensory integration, hence integration of the (seen) tool and the (felt) body controlling it (Kirsch & Kunde, 2023). Tools which move spatially incompatibly to the body come with lower levels of ownership in terms of explicit report (Liesner & Kunde, 2020) and implicit measures in terms of judged spatial differences between body and tool (Liesner et al., 2020a, b). This suggests that multisensory integration takes place to a lesser extent with incompatible as compared with compatible tool-transformations. A candidate reason for such lack of multisensory integration might be that the somatosensory representation of the body becomes downregulated to an extent that no integration with the visual representation of the body can take place. The present results, however, do not support this hypothesis, thus prompting more research regarding the reasons for reduced ownership experience of spatially incompatible moving tools.

Despite being unexpected, our results carry positive implications for applied contexts. While planning a tool movement does reduce tactile sensitivity compared with not doing so, this reduction does not appear to depend on the type of tool-transformation (i.e., whether it is compatible or incompatible to the operating effector). This finding is reassuring when thinking of situations in which incompatible tools are used while tactile perception is highly important, such as in minimal-invasive surgery. Moreover, the tactile modality appears suitable for conveying information (such as warnings) to users, regardless of the tool-transformation in which they engage. That said, the present and prior research (see Kunde et al., 2012) suggest that providing tactile (or auditory) information during action should be avoided to ensure it is processed.

### Limitations

Although our measures for tactile sensitivity proved eligible to reliably detect movement-induced suppression, perhaps they were not sensitive enough to detect the more subtle neglect during incompatible tool-transformations, and alternative procedures might provide different results. However, we find it most likely that tactile sensitivity might simply not reflect the neglect of critical body-related effect codes. Sensorimotor integration research (Debats et al., 2017; Debats & Heuer, 2018; Heuer & Rapp, 2012; Knoblich & Kircher, 2004; Ladwig et al., 2012; Müsseler & Sutter, 2009; Sutter et al., 2008) and the mirror drawing performance of a deafferented patient (Lajoie et al., 1992) clearly point towards the body as the cause of performance costs during incompatible tool-transformations. It is conceivable that the neglect of certain effect codes to overcome those costs pertains primarily to proprioception, while sensitivity to tactile events remains unaffected. Testing this hypothesis would require proprioceptive stimulation protocols during movement preparation, which we did not implement here.

Alternatively, sensorimotor integration paradigms that rely on subjects’ evaluation of previously produced bodily movements offer a means to assess the accessibility of proprioceptive action effects (e.g., Debats et al., 2017; Debats & Heuer, 2018; Sutter et al., 2008). Typically, these movements are transformed into visual effects that are subtly perturbed in size or direction, leading participants to adjust their estimation of their movements towards the altered visual feedback. When the effect on the screen is additionally inverted, these aftereffects have been shown to be reduced (Ladwig et al., 2012). However, such experimental designs are constraint by the need for participants to closely monitor their hand movements. Ideally, future studies need to extract measures of proprioceptive neglect without directing subjects’ attention towards the characteristics of their movements.

## Supplementary Information

Below is the link to the electronic supplementary material.

Supplementary file1 (DOCX 1648 KB)

## Data Availability

All experiments reported in this manuscript were preregistered on the Open Science Framework: (Experiment 1: https://osf.io/2c9be/?view_only=b67fda8b39dd4901aa9f1e851803d3b3, Experiment 2: https://osf.io/jn5rx/?view_only=d272e5461dad48889fc06c78da661cc8, Experiment 3: https://osf.io/jfzxp/?view_only=a4ce109d3b3244a1aa5a9b1572baef77, and Experiment 4: https://osf.io/m3w48/?view_only=d067189eb393451e9cb584639ba99457), where all materials, data, and scripts used in the experiments and their analyses are available through a link in the *Open Practice Resources – Data* section and stored in the *Files* tab of the respective projects.
