# Supplementary material for: Spatially incompatible tool use does not induce tactile neglect
Source: Atten Percept Psychophys. 2025 Dec 2;88(1):17. doi: 10.3758/s13414-025-03170-y (PMC12672823; doi:10.3758/s13414-025-03170-y)
Supplement: Supplementary file 1 — Supplementary file1 (DOCX 1648 KB) [file 13414_2025_3170_MOESM1_ESM.docx]

Supplementary Material for

Spatially incompatible tool use does not induce tactile neglect

in

Attention, Perception, & Psychophysics

Yanick Kloß^1*^ & Wilfried Kunde^1^

^1^University of Würzburg, Department of Psychology (III), Germany

*Corresponding author. E-mail: yanick.kloss@uni-wuerzburg.de

**Analysis of Experiment 1 on a Reduced Sample**

Since we preregistered to control for participation in a pilot study, we ran the analyses of Experiment 1 on a reduced sample without the respective participants. The results are summarized in Table S4 and mirrored those for the entire sample in terms of performance and sensitivity in the three conditions with the exception that for d’, the interaction between tool-transformation condition and stimulation timing was no longer significant.

**Analysis of Detection Thresholds in Experiment 1**

We computed psychometric functions per subject and condition using the quickpsy package in R. Detection thresholds represent the intensity level where a participant is expected to detect a stimulation 50% of the times. However, only for 27 participants, all detection thresholds were within the intensity range we presented. Many of the remaining participants detected even the weakest stimulation in more than 50% of the trials in at least one of the conditions, suggesting that intensities were too strong to compute reliable detection thresholds (n=14). Others failed to detect even the strongest stimulation in more than 50% of the trials, indicating that intensities were too weak (n=5). Thresholds of the remaining eight participants suggested that intensities were too weak in the baseline condition, but too strong in at least one of the two movement conditions, mirroring the large general tactile suppression effect in d’. We decided to tackle interindividual and intraindividual sensitivity differences reflected in these observations in Experiment 3 and to exclude any subjects with at least one threshold outside the intensity range for this analysis.

The ANOVA revealed only a main effect of tool-transformation condition, *F*(2,42) = 13.82, *p* < .001, η_p_² = .40. We computed pairwise post-hoc *t*-tests to compare detection thresholds at the different stimulation time points in the three blocks. Detection thresholds were significantly lower in the baseline compared to both compatible and incompatible tool-transformation at both stimulation times (all *p* < .005). Thresholds were only descriptively lower in compatible compared to incompatible trials before movement onset (2.69 vs. 2.75), *t*(21) = 0.26, *p* > .999, *d_z_* = 0.06, *BF*_10_ = 0.23, but not after movement onset (2.83 vs. 2.77), *t*(21) = 0.25, *p* > .999, *d_z_* = 0.05, *BF*_10_ = 0.23.

**Analysis of Response Times and Accuracy as a Function of Tactile Stimulation (Timing)**

As a reviewer correctly noted, it is intriguing to investigate if receiving a tactile stimulus shortly before the initiation of a movement affects performance in any way. We computed 2 * 3 ANOVAs with the factors tool-transformation (compatible vs. incompatible) and stimulation timing (no stimulation vs. stimulation before movement onset vs. stimulation after movement onset) for Experiments 1,3, and 4, and a 2*2 ANOVA with the factors tool-transformation and stimulation timing (before vs. after movement onset) for Experiment 2. We analyzed accuracy across all experiments, but response times only for Experiments 3 and 4.

Unfortunately, we did not log the exact timing of stimulations in error trials in Experiments 1 and 2. Here, we computed the ANOVAs using the original assignment of trials to early and late stimulations that was based on the anticipated movement onset. For Experiments 3 and 4, we ran the analyses using both this original classification and the classification based on the actual movement onsets.

In Experiment 1, the ANOVA did not reveal any effects of tactile stimulation on accuracy. In Experiment 2, there was a main effect of stimulation timing, *F*(1,44) = 9.18, *p* = .004, η_p_² = .17. In compatible trials, accuracy was larger when participants received a tactile stimulus shortly before initiating their movement, *t*(45) = 3.68, *p* < .001, *d*_z_ = 0.55.

In Experiment 3, the analysis of error rates did not show any effects of stimulation. When including the stimulation’s temporal relationship with the actual instead of the anticipated movement onset to analyze response times, not only a main effect of tool-transformation, *F*(1,52) = 5.20, *p* = .027, η_p_² = .09, but also a large main effect of stimulation timing emerged, *F*(2,104) = 198.26, *p* <.001, η_p_² = .79. Note however that using the actual movement onset to assign trials to a stimulation timing condition meant that we had already grouped slower responses into the early and faster responses into the late stimulation condition. Indeed, the effect of stimulation timing completely vanished when computing the ANOVA with the originally assigned timing condition, *F*(2,102) = 1.08, *p* = .343, η_p_² = .02, suggesting that the effect was primarily driven by the few trials where the movement onset was earlier or later than expected, and not by an effect of the stimulation itself.

In Experiment 4, The ANOVA on accuracy revealed an interaction between compatibility and stimulation, *F*(2,88) = 11.50, *p* < .001, η_p_² = .21. In compatible tool-transformation trials, accuracy was significantly reduced when a stimulus was applied shortly before movement onset compared to trials where it was applied after movement onset, *t*(47) = 4.11, *p* < .001, *d_z_* = 0.59, and trials where participants received no stimulation at all, *t*(47) = 3.63, *p* = .002, *d_z_* = 0.52. These results could be replicated when categorizing the stimulation timing based on the anticipated movement onset, but they stand in contrast to the results in Experiment 1 and should be therefore taken with great caution. Regarding response times, a large effect of stimulation (timing) seemed to appear when taking the actual movement onset as a reference for the timing, *F*(2,98) = 260.54, *p* < .001, η_p_² = .84. As in Experiment 3, it vanished when using the original timing categories, *F*(2,100) = 1.65, *p* = .197, η_p_² = .03.

Overall, these results do not provide conclusive evidence that receiving a tactile stimulus at the effector before movement onset affects performance in one way or the other. However, our studies are certainly not ideal to investigate this, and our results should not be taken as evidence against an effect, either.

**Supplementary Figures**

**Figure S1**

*Mean stimulation timing relative to movement onset for each subject and timing condition.*


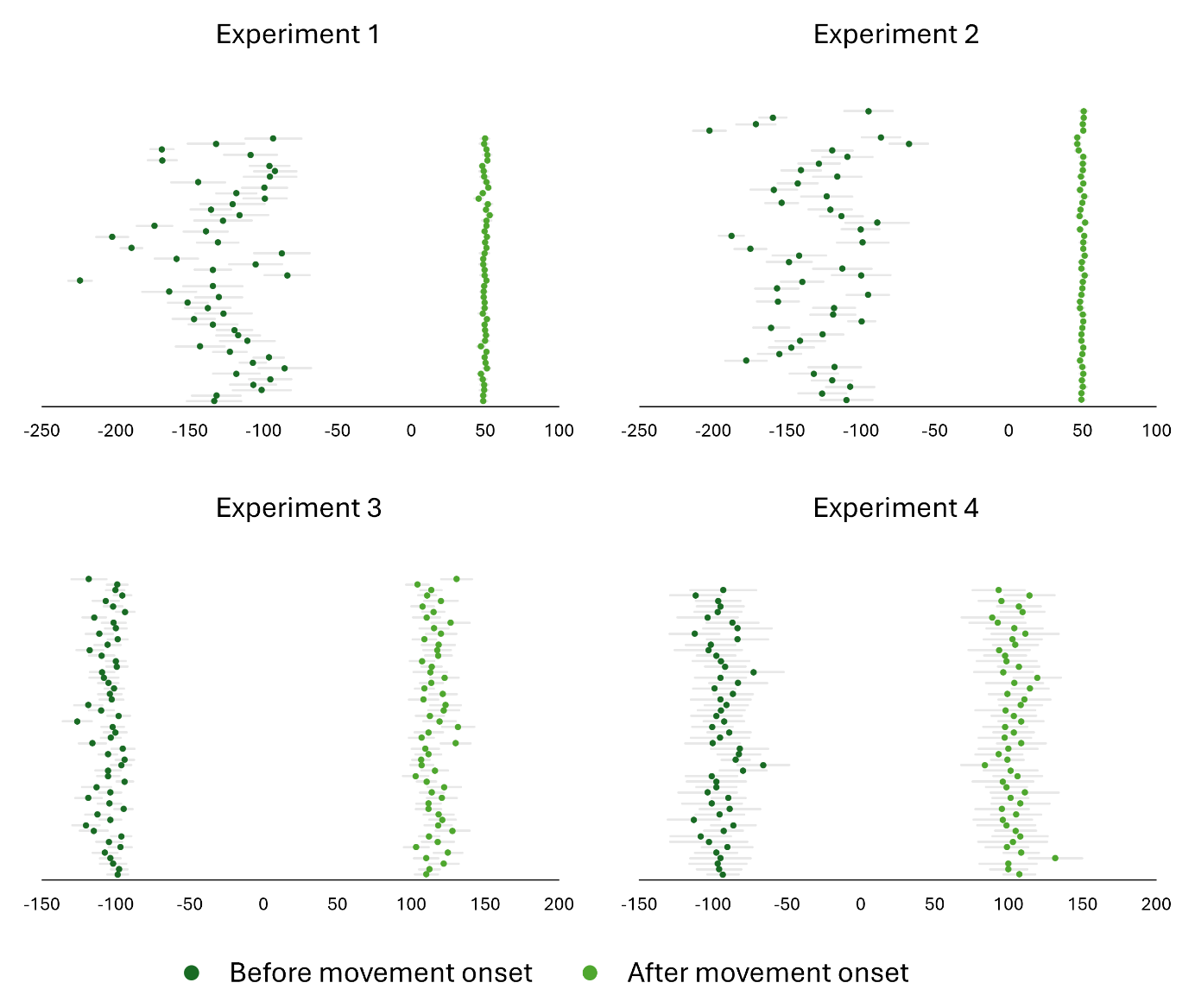


*Note.* Error bars represent 95% confidence intervals. For Experiment 1 and 2, means and CIs were computed on movement trials only, since the stimulation timing in the baseline condition did not depend on participants’ responses, but varied only within the frame rate of the computer.

**Figure S2**

*Exemplary psychometric plot of an excluded participant in Experiment 1 displaying no relationship between stimulus intensity and detection probability.*


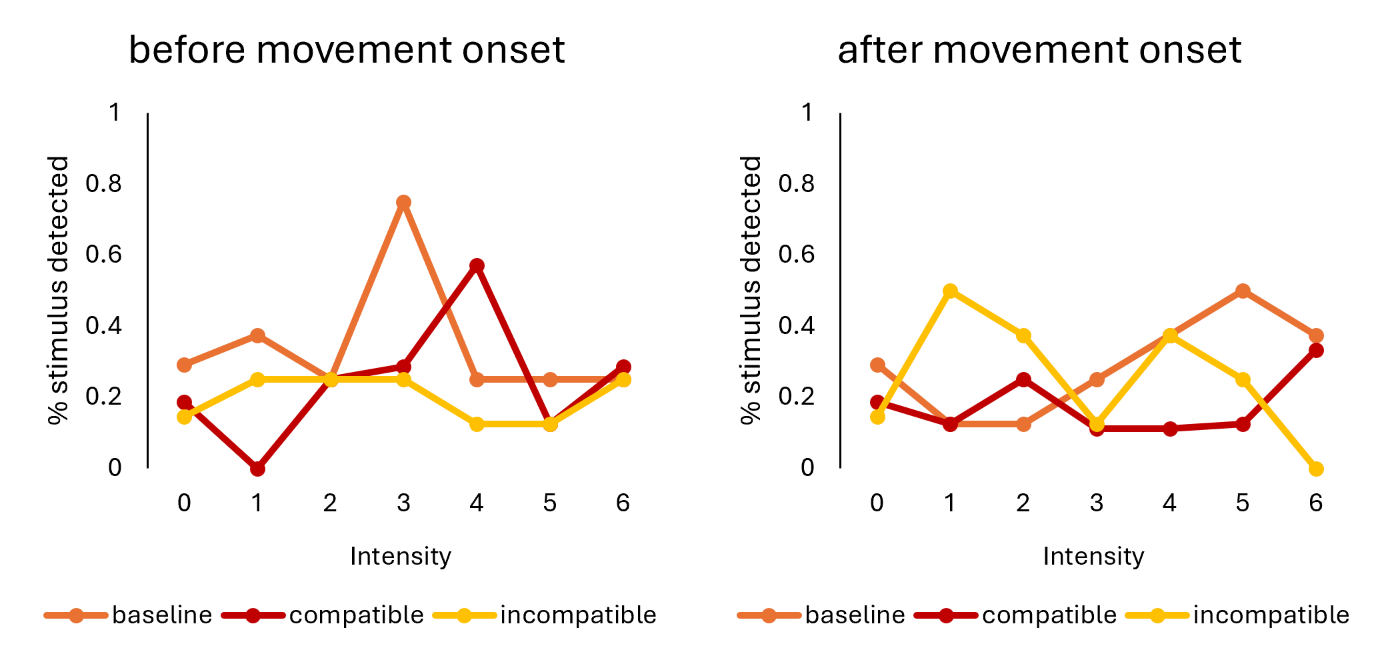


*Note.* Intensity 0 represents noise trials without any stimulation.

**Figure S3**

*Tactor positioning in Experiments 1 (A), 2 (B), 3 (A), and 4 (C).*

**
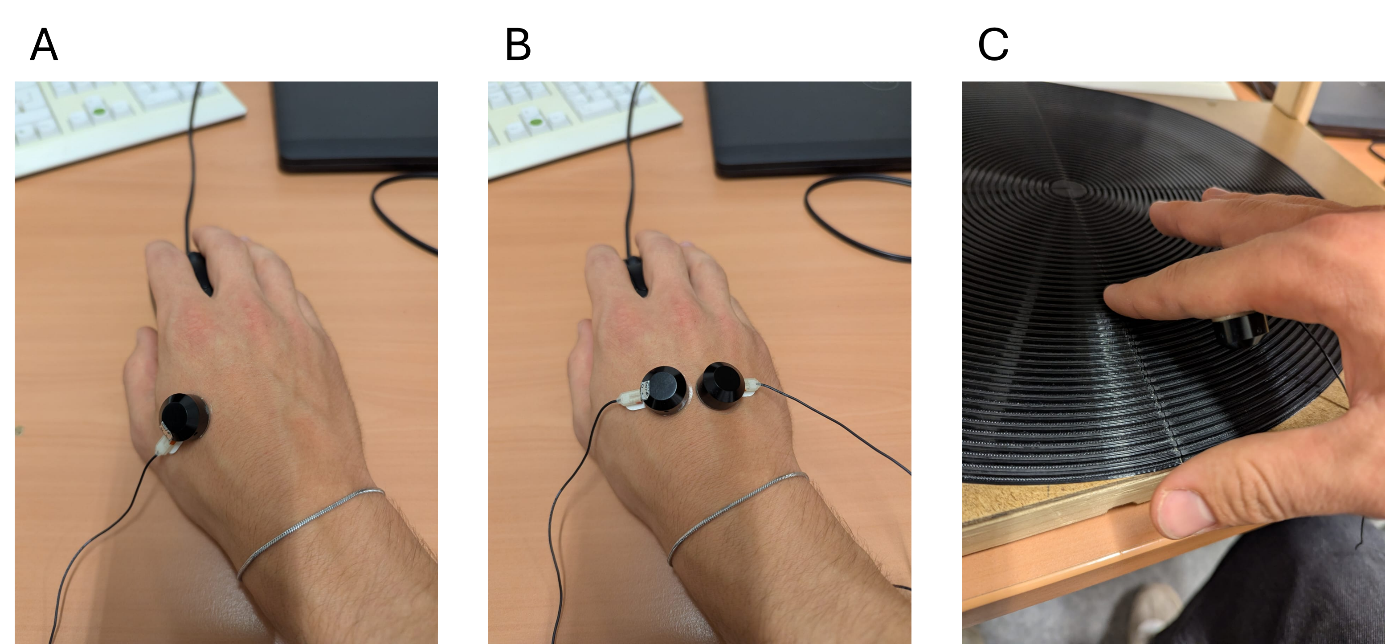
**

**Figure S4**

*Visualization of the setup of Experiment 4.*


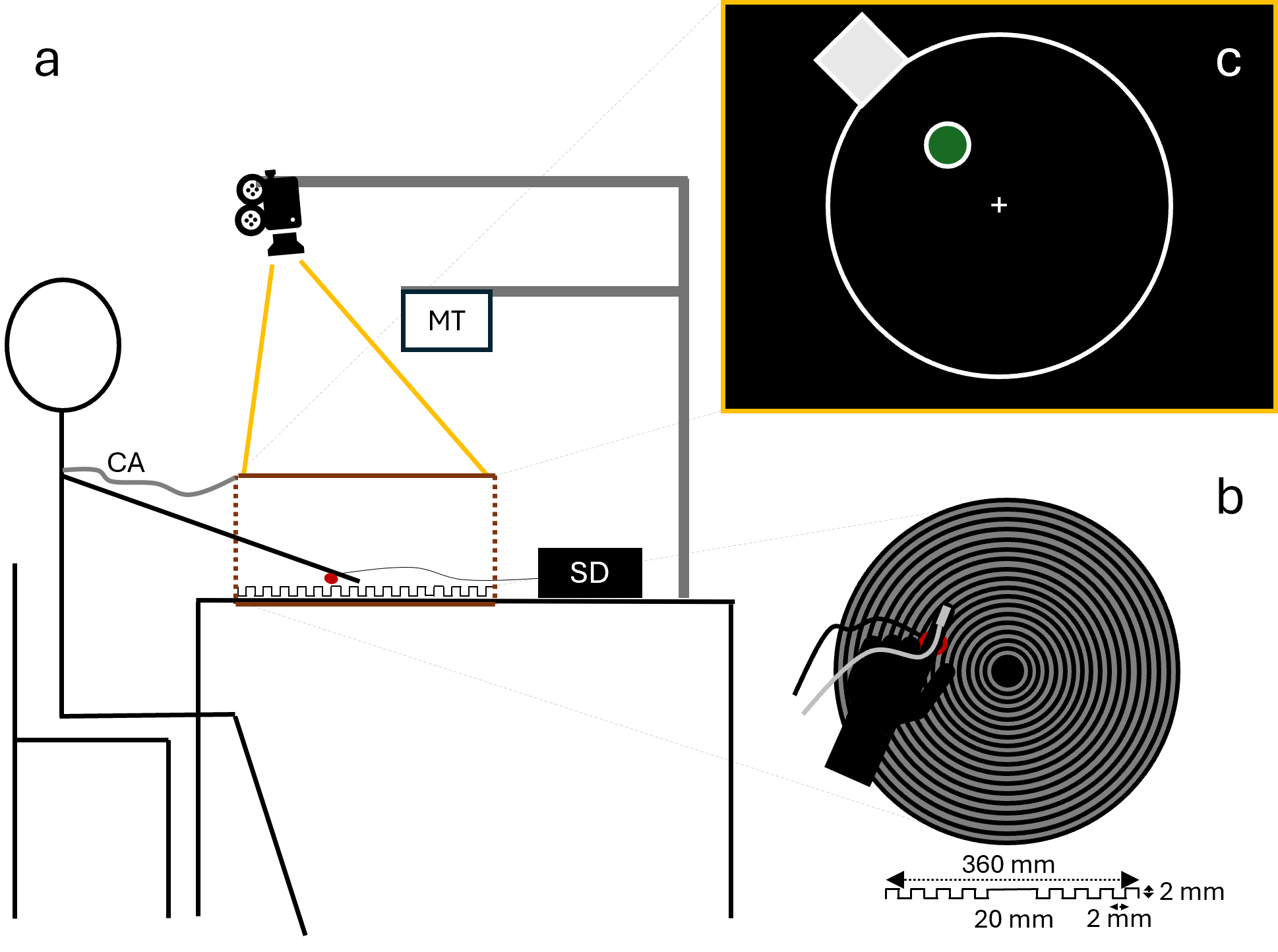


*Note.* a) Visualization of the basic setup (MT = motion tracking device; SD = stimulation device, CA = cape). b) Polylactide surface with ridges and grooves, participant’s hand with motion tracking sensor (grey) and tactor (red). c) Visual image corresponding to the position of the hand in b) in a compatible trial.

**Figure S5**

*Exemplary movement trajectory in an incompatible trial that was discarded as an error because the participant initiated a movement in the wrong direction before correcting it*


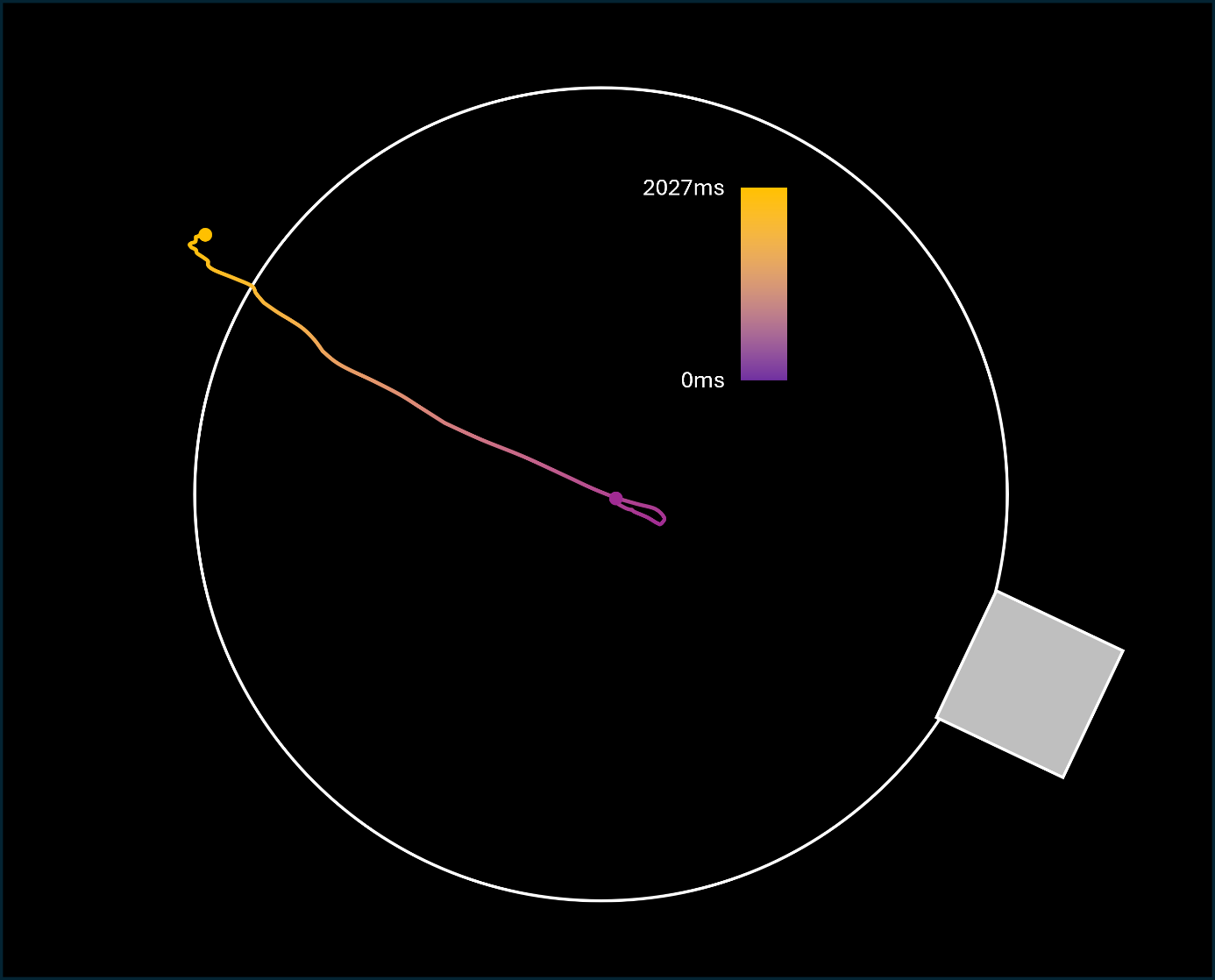


**Table S1**

*ANOVA effects for c and β across Experiments 1-4*

|  | Measure | ANOVA Effect | *df*_num,den_ | *F* | *p* | η_p_² |
| --- | --- | --- | --- | --- | --- | --- |
| Exp 1 | *c* | Transformation | 2,96 | 11.59 | <.001 | .19 |
|  |  | Timing | 1,48 | 2.24 | .141 | .05 |
|  |  | Interaction | 2,96 | 5.09 | .008 | .10 |
|  | β | Transformation | 2,96 | 0.95 | .392 | .02 |
|  |  | Timing | 1,48 | 1.50 | .227 | .03 |
|  |  | Interaction | 2,96 | 0.18 | .834 | <.01 |
| Exp 2 | *c* | Transformation | 2,88 | 0.77 | .446 | .02 |
|  |  | Timing | 1,44 | 0.26 | .512 | .01 |
|  |  | Interaction | 2,88 | 0.29 | .750 | .01 |
|  | β | Transformation | 2,88 | 2.00 | .142 | .04 |
|  |  | Timing | 1,44 | 0.75 | .391 | .02 |
|  |  | Interaction | 2,88 | 0.76 | .472 | .02 |
| Exp 3 | *c* | Transformation | 1,52 | 0.02 | .904 | <.01 |
|  |  | Timing | 1,52 | 10.79 | .002 | .17 |
|  |  | Interaction | 1,52 | 0.17 | .070 | .06 |
|  | β | Transformation | 1,54 | 0.52 | .476 | <.01 |
|  |  | Timing | 1,54 | 12.02 | .001 | .18 |
|  |  | Interaction | 1,54 | 0.35 | .556 | <.01 |
| Exp 4 | *c* | Transformation | 1,49 | 0.93 | .393 | .02 |
|  |  | Timing | 1,49 | 0.06 | .815 | <.01 |
|  |  | Interaction | 1,49 | 0.23 | .631 | .01 |
|  | β | Transformation | 1,52 | 0.02 | .893 | <.01 |
|  |  | Timing | 1,52 | 0.77 | .384 | .02 |
|  |  | Interaction | 1,52 | 4.09 | .048 | .07 |

**Table S2**

*Intensity levels in Experiment 3*

|  | Level distance | 0.33 | 0.67 | 1.00 | 1.33 | 1.67 | 2.00 | 2.33 | 2.67 | 3.00 | 3.33 |
| --- | --- | --- | --- | --- | --- | --- | --- | --- | --- | --- | --- |
| Minimum intensity level | 1 | 6.67 | 6.67 | 6.67 | 6.67 | 6.67 | 6.67 | 6.67 | 6.67 | 6.67 | 6.67 |
|  | 2 | 7.00 | 7.33 | 7.67 | 8.00 | 8.33 | 8.67 | 9.00 | 9.33 | 9.67 | 10.00 |
|  | 3 | 7.33 | 8.00 | 8.67 | 9.33 | 10.00 | 10.67 | 11.33 | 12.00 | 12.67 | 13.33 |
|  | 4 | 7.67 | 8.67 | 9.67 | 10.67 | 11.67 | 12.67 | 13.67 | 14.67 | 15.67 | 16.67 |
|  | 5 | 8.00 | 9.33 | 10.67 | 12.00 | 13.33 | 14.67 | 16.00 | 17.33 | 18.67 | 20.00 |
|  | 6 | 8.33 | 10.00 | 11.67 | 13.33 | 15.00 | 16.67 | 18.33 | 20.00 | 21.67 | 23.33 |
|  | 7 | 8.67 | 10.67 | 12.67 | 14.67 | 16.67 | 18.67 | 20.67 | 22.67 | 24.67 | 26.67 |
| Maximum intensity level | 8 | 9.00^a^ | 11.33^a^ | 13.67^a^ | 16.00^a^ | 18.33^a^ | 20.67^a^ | 23.00^a^ | 25.33^a^ | 27.67^a^ | 30.00^a^ |
|  | Intensity range | 1 | 2 | 3 | 4 | 5 | 6 | 7 | 8 | 9 | 10 |
|  | *n* Subjects | 0 | 0 | 2 | 2 | 5 | 5 | 3 | 7 | 2 | 29 |

*Note.* All intensity levels used in the pretest, practice, and main experiment are displayed in µM peak-to-peak displacement of the vibrating element. ^a^ Intensities presented in the pretest. If, for instance, the stimulation with a peak-to-peak displacement of 16.00 µM (intensity range -1) was the lowest intensity detected in more than 78% of correct trials, intensity range 4 was chosen for the main experiment.

**Table S3**

*Intensity levels in Experiment 4*

|  | Level distance | 0.4 | 0.8 | 1.2 | 1.6 | 2.0 | 2.4 | 2.8 | 3.2 | 3.6 | 4.0 | 4.4 | 4.8 |
| --- | --- | --- | --- | --- | --- | --- | --- | --- | --- | --- | --- | --- | --- |
| Minimum intensity level | 1 | 6.67 | 6.67 | 6.67 | 6.67 | 6.67 | 6.67 | 6.67 | 6.67 | 6.67 | 6.67 | 6.67 | 6.67 |
|  | 2 | 7.07 | 7.47 | 7.87 | 8.27 | 8.67 | 9.07 | 9.47 | 9.87 | 10.27 | 10.67 | 11.07 | 11.47 |
|  | 3 | 7.47 | 8.27 | 9.07 | 9.87 | 10.67 | 11.47 | 12.27 | 13.07 | 13.87 | 14.67 | 15.47 | 16.27 |
|  | 4 | 7.87 | 9.07 | 10.27 | 11.47 | 12.67 | 13.87 | 15.07 | 16.27 | 17.47 | 18.67 | 19.87 | 21.07 |
|  | 5 | 8.27 | 9.87 | 11.47 | 13.07 | 14.67 | 16.27 | 17.87 | 19.47 | 21.07 | 22.67 | 24.27 | 25.87 |
|  | 6 | 8.67 | 10.67 | 12.67 | 14.67 | 16.67 | 18.67 | 20.67 | 22.67 | 24.67 | 26.67 | 28.67 | 30.67 |
|  | 7 | 9.07 | 11.47 | 13.87 | 16.27 | 18.67 | 21.07 | 23.47 | 25.87 | 28.27 | 30.67 | 33.07 | 35.47 |
| Maximum intensity level | 8 | 9.47^a^ | 12.27^a^ | 15.07^a^ | 17.87^a^ | 20.67^a^ | 23.47^a^ | 26.27^a^ | 29.07^a^ | 31.87^a^ | 34.67^a^ | 37.47^a^ | 40.27^a^ |
|  | Intensity range | -2 | -1 | 1 | 2 | 3 | 4 | 5 | 6 | 7 | 8 | 9 | 10 |
|  | *n* Subjects |  |  | 9 | 15 | 21 | 2 | 2 | 0 | 0 | 2 | 0 | 2 |

*Note.* All intensity levels used in the pretest, practice, and main experiment are displayed in µM peak-to-peak displacement of the vibrating element. ^a^ Intensities presented in the pretest. If, for instance, the stimulation with a peak-to-peak displacement of 12.27 µM (intensity range -1) is the lowest intensity detected in more than 74% of correct trials, intensity range 2 is chosen for the main experiment.

**Table S4**

*Tactile suppression and body-related neglect effects in Experiment 1 for the entire sample and a sample where those participants who had already taken part in a pilot study were excluded*

|  |  | Effect | | | | | | | |  |  |  |  |  |
| --- | --- | --- | --- | --- | --- | --- | --- | --- | --- | --- | --- | --- | --- | --- |
|  |  | Tactile suppression  Δbaseline-compatible | | | | Body-related neglect  Δcompatible-incompatible | | | |  |  |  |  |  |
|  |  | Stimulation timing | | | | | | | |  |  |  |  |  |
|  |  | Pre  movement onset | | Post  movement onset | | Pre  movement onset | | Post  movement onset | |  |  |  |  |  |
|  | Measure | M | BF_10_ | M | BF_10_ | M | BF_10_ | M | BF_10_ | ANOVA Effect | *df*_num,den_ | *F* | *p* | η_p_² |
| Large sample | d’ | 0.75 | 11.1^E+6^ | 0.93 | 15.0^E+8^ | 0.10 | 0.42 | -0.01 | 0.16 | Transformation | 2,96 | 66.69 | <.001 | 0.58 |
|  |  |  |  |  |  |  |  |  |  | Timing | 1,48 | 2.24 | .141 | 0.05 |
|  |  |  |  |  |  |  |  |  |  | Interaction | 2,96 | 5.09 | .008 | 0.10 |
|  | Thresholds | -0.91 | 125.51 | -1.16 | 56.4^E+2^ | -0.07 | 0.23 | 0.07 | 0.23 | Transformation | 2,42 | 13.82 | <.001 | 0.40 |
|  |  |  |  |  |  |  |  |  |  | Timing | 1,21 | 0.05 | .824 | <0.01 |
|  |  |  |  |  |  |  |  |  |  | Interaction | 2,42 | 1.23 | .303 | 0.06 |
| Reduced sample | d’ | 0.71 | 25.2^E+4^ | 0.86 | 47.8^E+5^ | 0.13 | 0.65 | 0.08 | 0.27 | Transformation | 2,76 | 53.84 | <.001 | 0.59 |
|  |  |  |  |  |  |  |  |  |  | Timing | 1,38 | 0.16 | .688 | <0.01 |
|  |  |  |  |  |  |  |  |  |  | Interaction | 2,76 | 2.85 | .064 | 0.01 |
|  | Thresholds | -0.79 | 51.48 | -0.99 | 93.4^E+1^ | -0.12 | 0.26 | -0.02 | 0.24 | Transformation | 2,36 | 9.82 | <.001 | 0.35 |
|  |  |  |  |  |  |  |  |  |  | Timing | 1,18 | 0.23 | .640 | 0.01 |
|  |  |  |  |  |  |  |  |  |  | Interaction | 2,36 | 0.75 | .479 | 0.04 |

*Note.* Positive values for d’ and negative values for detection thresholds indicate the existence of the respective effect. All Bayes Factors were computed with a prior distribution of $\frac{\sqrt{2}}{2}$. Greenhouse-Geisser correction was used to account for sphericity-violations in all four experiments.
